# Consciousness and the Dimensionality of DOC Patients via the Generalized Ising Model

**DOI:** 10.3390/jcm9051342

**Published:** 2020-05-04

**Authors:** Pubuditha M. Abeyasinghe, Marco Aiello, Emily S. Nichols, Carlo Cavaliere, Salvatore Fiorenza, Orsola Masotta, Pasquale Borrelli, Adrian M. Owen, Anna Estraneo, Andrea Soddu

**Affiliations:** 1 Department of Physics and Astronomy, Western University, London ON N6G2V4, Canada; enicho4@uwo.ca (E.S.N.); asoddu@uwo.ca (A.S.); 2 Brain and Mind Institute, Western University, London ON N6A57, Canada; aowen6@uwo.ca; 3 School of Psychological Sciences and Turner Institute for Brain and Mental Health, Monash University, Melbourne, VIC 3800, Australia; 4 IRCCS SDN, Via E. Gianturco 113, 80143 Naples, Italy; maiello@sdn-napoli.it (M.A.); ccavaliere@sdn-napoli.it (C.C.); pasquale.borrelli@synlab.it (P.B.); 5 Clinical Scientific Institute Maugeri; Telese Terme Center; 82037 Telese Terme, Italy; sasott8@gmail.com (S.F.); orsola.masotta@gmail.com (O.M.); aestraneo@gmail.com (A.E.); 6 Department of Psychology, Western University, London ON N6A5C2, Canada; 7 Department of Physiology and Pharmacology, Western University, London ON N6A5C1, Canada; 8 Neurology Unit, SM della Pietà General Hospital, 80035 Nola, Italy

**Keywords:** disorder of consciousness, generalized Ising model, dimensionality of the brain, structural connectome, functional connectivity

## Abstract

The data from patients with severe brain injuries show complex brain functions. Due to the difficulties associated with these complex data, computational modeling is an especially useful tool to examine the structure–function relationship in these populations. By using computational modeling for patients with a disorder of consciousness (DoC), not only we can understand the changes of information transfer, but we also can test changes to different states of consciousness by hypothetically changing the anatomical structure. The generalized Ising model (GIM), which specializes in using structural connectivity to simulate functional connectivity, has been proven to effectively capture the relationship between anatomical structures and the spontaneous fluctuations of healthy controls (HCs). In the present study we implemented the GIM in 25 HCs as well as in 13 DoC patients diagnosed at three different states of consciousness. Simulated data were analyzed and the criticality and dimensionality were calculated for both groups; together, those values capture the level of information transfer in the brain. Ratifying previous studies, criticality was observed in simulations of HCs. We were also able to observe criticality for DoC patients, concluding that the GIM is generalizable for DoC patients. Furthermore, dimensionality increased for the DoC group as compared to healthy controls, and could distinguish different diagnostic groups of DoC patients.

## 1. Introduction

Advances in life-saving medical technology have dramatically increased survival rates for comatose patients after severe brain injury. However, many of these patients will be diagnosed with a prolonged disorder of consciousness (pDoC) [1,2], a state in which they show disruption to their awareness of themselves and/or their environment. The pDoCs fall into three sub-categories: (1) the vegetative state/unresponsive wakefulness syndrome (VS/UWS), in which a patient is wakeful (i.e., the patient may open their eyes) but unaware (i.e., the patient may perform reflexive, non-intentional behaviours, but shows no evidence of brain activity associated with cognitive function); (2) the minimally conscious state minus (MCS−), in which patients show reproducible minimal evidence of awareness, such as visual pursuit or fixation, orientation to noxious stimuli, and vocalizations or contingent motor and affective responses, although their presence is inconsistent; and (3) the MCS plus (MCS+), in which patients show more complex volitional responses (such as command following, intelligible verbalization, or gestural or verbal yes or no responses) [3,4]. Additionally, the patient is classified as emergence from minimally conscious state (EMCS) when he/she is able to communicate or show proper functional object use [4]. In each of these conditions, the clinical assessment of awareness through behavioural tools can be extremely challenging and sometimes inconsistent between assessments, even when a standardized scale such as the Coma Recovery Scale [5] is administered [6]. Even though there are standardized tests to evaluate the state of the patients, the misdiagnosis rates using these scales are relatively high, which leaves room for the development of advanced and robust measures for assessing the state of consciousness of patients [7,8]. One emerging theory, the integrated information theory (IIT), is used to calculate a measure of complexity (consciousness) of a system (brain) and also provides promising results for the development of tools to assess the consciousness of non-responsive patients [9,10,11]. Moreover, determining whether a patient’s condition will improve remains largely subjective because of the lack of reliable prognostic indices [12,13,14]. Additionally, determining the clinical evolution of individuals with DoC has not yet been established, as 40% of them do not survive past the first two weeks due to withdrawal of life support [15,16,17]. In those patients that remain on life support, approximately half go on to recover some degree of awareness [15].

One source of prognostic information may arise from the link between structure and function in the brain. In healthy individuals, brain structure is similar. Although individual differences exist, the various segments of white matter that connect near and distant areas of the brain can easily be identified. This network of white matter is the brain’s structural connectivity. In turn, the function of the brain depends on these connections, which are responsible for transmitting information between regions of the cortex. The interaction of different brain areas can also be studied by way of their functional connectivity: How much do the activity in two areas of the brain correlate or anti-correlate. Several functional brain networks have been identified, such as the default mode network (DMN), which describes the brain at rest, and the executive control network (ECN), which describes the brain during tasks requiring executive function. The functional connectivity can be altered in several pathologies, such as Alzheimer’s disease [18], Parkinson’s disease [19], and depression [20].

Although functional connectivity can be used to identify pathologies in individuals with largely regular brain anatomy [20,21], its usefulness is complicated in the case of patients with severe brain injury. In such patients, brain structure has often been greatly altered, and no longer resembles a normal template. Brain regions also no longer interact normally, and it is difficult to determine what sort of internal cognition is arising from the observed functional connectivity. For these reasons, comparison of functional connectivity to healthy individuals may not be productive, and conclusions about a patient’s awareness, cognitive function, and prognosis are difficult to draw.

Because of the difficulties associated with data from patients with severe brain injury, computational modeling is an especially useful tool when examining the structure–function relationship in these populations. Such models aim to describe information flow amongst the regions of a complex system and can simulate both healthy and injured neural systems [22]. Using computational models, we can test the predictions of different theories of information transfer in the brain and make causal inferences about empirically observed behaviour. Specifically, in patients with a disorder of consciousness (DoC), such simulations can aid in determining what sort of residual cognition remains based on how information is flowing between different regions and how it has been disrupted. It could also lead to the development of treatment schemes to improve the patients’ brain function.

Previous work has shown that the generalized Ising model (GIM) can effectively capture the connection between anatomical structures and spontaneous fluctuations of the brain, as captured by functional magnetic resonance imaging (fMRI) [22,23,24,25]. The GIM, based on the classical Ising model originally used to describe ferromagnetic behaviour, simulates the blood-oxygenation-level-dependent (BOLD) signal measured using fMRI. Each region of the brain is modeled by a spin site within a lattice, with connections between lattice sites weighted according to the empirical structural connectivity between brain regions. Empirical BOLD signals greater than a certain threshold can be represented by up spins, and below the threshold can be represented by down spins. Using this model, information flow throughout the brain has been successfully simulated in a healthy system [22]. To date, the GIM has only been used to model healthy brains. The present study aimed to implement this model in both healthy controls as well as a sample of brain-injured patients with DoC. To do this, we first generated the empirical correlation matrix of the BOLD time series in 84 brain regions. We then used the GIM to generate time series using each subject’s structural connectivity and ran simulations to determine the temperature that led to the highest correlation between the simulated and empirical data.

Finally, we also sought to compare the model parameters between controls and patients with DoC. Recent work has shown that the generalized Ising model at criticality can predict spontaneous brain activity, or functional connectivity, in healthy individuals. In this study, we attempted to determine whether the GIM at criticality can be extended to predict functional connectivity of the brain in patients with DoC, who often have widespread structural brain damage.

Recently, using the structural connectome, the healthy brain was found to be two-dimensional [22]. To define the dimensionality of the brain using the structural connectome and the rate of information transfer, we introduced a space where the distance between two regions of the brain is measured by the strength of the connectome rather than the physically measurable distance. Choice of this definition agrees with the level of information transfer in the brain based on the structural connectome. By introducing the new measure of the distance, we calculated the decay of information transfer with respect to the newly defined distance (the number of fibres between each pair of regions in the brain). Dimensionality represents the rate of decay in information transfer with respect to the distance, in that a higher dimensionality implies a faster rate of decay. Dimensionality is one parameter of the GIM. Its calculation depends on the distance between two regions, measured here by the number of fibres connecting those regions. We then determined the functional correlation between all the regions at the same distance and plotted the results in order to calculate the dimensionality. In healthy participants, the brain was highly correlated, showing strong relationships between many regions [22]. The dimensionality of the brain in patients with DoC, however, has not yet been determined.

Thus, a further goal of this study was to determine if this parameter remains the same in patients with DoC. Additionally, if dimensionality is different in these patients, does it change between clinical categories of DoC or does it remain constant within DoC as a whole? That is, can we distinguish between different levels of consciousness using dimensionality? Based on research in healthy participants and the assertion that brain activity relies on structure and the connections between different areas, we hypothesized that functional connectivity will be tied to brain structure in patients with DoC to the same degree. However, we predicted that dimensionality will increase in patients relative to controls, due to damaged structural connectivity. Specifically, we predicted that a higher distance between regions (i.e., a lower number of fibers connecting them) will lead to a faster decay in functional correlation, potentially functioning as a diagnostic factor in these patients.

## 2. Experimental Section

### 2.1. Materials

#### 2.1.1. Participants

We screened a series of individuals with prolonged (i.e., time post injury greater than 28 days) DoC from January 2016 to July 2018 admitted to a dedicated Neurorehabilitation Unit of ICS Maugeri, Telese Terme, Italy. For this study, we enrolled patients fulfilling standardized clinical criteria for VS/UWS or MCS [2]. Patients with the latter diagnosis were further classified as being in MCS+ or MCS− according to recent criteria [3]. We excluded from the study patients with: (i) severe pathologies independent from the brain injury (e.g., premorbid history of psychiatric or neurodegenerative diseases); (ii) mixed etiology (e.g., both traumatic and anoxic brain injury); (iii) non-stabilized and severe general clinical conditions; (iv) contra-indication for magnetic resonance imaging (MRI) (e.g., ferromagnetic aneurysm clips, pacemaker); (v) large brain damage (>50% of total brain volume), as stated by a certified neuroradiologist on computed tomography (CT) or MRI scan performed before the positron emission tomography (PET)/fMRI acquisition (patients with macroscopic traumatic brain damage, such as to distort normal anatomy, were excluded after a careful radiological evaluation); and (vi) motion parameters >3 mm in translation and 3 mm in rotation. Patients were also excluded if their clinical diagnosis had changed in the week preceding the neuroimaging acquisition.

From a sample of 29 severely brain-injured patients with DoC (Figure S1), we considered for PET/fMRI analysis a set of thirteen patients with pDoC (VS/UWS = 6; MCS− = 3; MCS+ = 4) due to severe traumatic (*n* = 3), vascular (*n* = 5), or anoxic (*n* = 5) brain injury, aged between 18 and 73. Detailed descriptions of patients’ clinical features are provided in Table 1.

#### 2.1.2. Controls

We also examined a group of twenty-five healthy participants, aged between 20 and 64 (13 female). Controls’ PET/MRI studies were carried out at St. Joseph’s hospital London, Ontario, using the same protocol and a similar PET/MRI instrumentation to the DoC patients’ scans.

#### 2.1.3. Ethics Statement

The study was approved by the local Ethics Committee of IRCCS Pascale (Protocol number: 3/15; 20/05/2015) and performed according to the ethical standards laid down in the 1964 Helsinki Declaration and its later amendments. Written informed consent was obtained from the legal guardian of patients in the ICS Maugeri Neurorehabilitation Unit. The study involving healthy controls was approved by the health science research ethics board of Western University and written informed consent was obtained from the participants before completing the study.

#### 2.1.4. Clinical Assessment of DoC Patients

One week before and one week after neuroimaging recording, all enrolled patients underwent at least five clinical evaluations, using the Italian version of the coma recovery scale-revised (CRS-R) [26], in order to confirm a stabilized clinical diagnosis of VS/UWS, MCS−, or MCS+ and to gather the best CRS-R total score. Patients’ consciousness level (measured by CRS-R total and sub-scores) was also assessed on the neuroimaging day. All behavioral assessments were performed by one skilled psychologist (OM).

#### 2.1.5. MRI Data Acquisition

MR images were acquired using high resolution 3T PET/MRI scans at St. Joseph’s Hospital, London, ON, Canada, for controls and at the IRCCS SDN, Naples, Italy, for the patients. Vacuumed pillows were used to minimize head movements within the scanner. The PET/MRI was acquired in the morning after customary nursing procedures. We also employed strategies to ensure patients’ best vigilance state by: (i) stopping possible sedative drugs (such as benzodiazepines) 15 h before scanning; (ii) administering CRS-R vigilance protocol [5] before PET/MRI acquisition and during the neuroimaging exam at the end of the first resting state of MRI acquisition; and (iii) monitoring eye openings by means of a video camera located in the MRI scanner. In case of appearance of clinical signs of possible drowsiness (i.e., persistence of eye closing), MRI acquisition was stopped, and the CRS-R vigilance protocol was re-administered.

The following MRI sequences were sequentially run:

I. First rs-fMRI acquisition (named “t1”) by a T2 *-weighted single-shot EPI sequence (voxel-size 4 × 4 × 4 mm^3^, repetition time (TR)/echo time (TE) = 1000/21.4 ms, flip angle = 82°, 480 time points, field of view (FOV) read = 256 mm, multiband factor = 2, distance factor = 0, TA = 8′06″).
II. Three-dimensional T1-weighted magnetization-prepared rapid acquisition gradient-echo sequence (MPRAGE, 240 sagittal planes, 256 × 214 mm field of view, voxel size 0.8 × 0.8 × 0.8 mm^3^, TR/TE/inversion time (TI) 2400/2.25/1000 ms, flip angle 8°, acquisition time (TA) = 6′18″)
III. Three-dimensional T2-weighted sequence (240 sagittal planes, 256 × 214 mm field of view, voxel size 0.8 × 0.8 × 0.8 mm^3^, TR/TE 3370/563 ms, TA = 6′46″).
IV. Three-dimensional fluid attenuation inversion recovery (FLAIR, 160 sagittal planes, 192 × 192 mm field of view, voxel size 1 × 1 × 1 mm3, TR/TE/TI 5000/334/1800 ms, TA = 6′42″).
V. Second rs-fMRI acquisition (named “t2”) by a T2 *-weighted single-shot EPI sequence (voxel-size 4 × 4 × 4 mm^3^, TR/TE = 1000/21.4 ms, flip angle = 82°, 480 time points, FOV read = 256 mm, multiband factor = 2, distance factor = 0, TA = 8′06″). The two rs-fMRI acquisitions (t1 and t2) were separated by a 30 min interval.
VI. Diffusion tractography (TR: 3,851, TE: 84.2 voxel: 2 mm^3^ isotropic, axial planes; 71 directions; *b* value max: 1500, matrix: 128 × 128 acquired both with Anterior-Posterior and Posterior-Anterior phase encoding).

##### Anatomical Data Preprocessing

The T1-weighted (T1w) image was corrected for intensity non-uniformity (INU) with N4BiasFieldCorrection [27], distributed with ANTs 2.2.0 [28], and used as the T1w reference throughout the workflow. The T1w reference was then skull-stripped with a Nipype implementation of the antsBrainExtraction.sh workflow (from ANTs), using OASIS30ANTs as the target template. Brain tissue segmentation of cerebrospinal fluid (CSF), white-matter (WM), and gray-matter (GM) was performed on the brain-extracted T1w using fast FMRIB software library (FSL 5.0.9, [29]). Brain surfaces were reconstructed using recon-all (FreeSurfer 6.0.1, [30]), and the brain mask estimated previously was refined with a custom variation of the method to reconcile ANTs-derived and FreeSurfer-derived segmentations of the cortical gray matter of Mindboggle [31]. Volume-based spatial normalization to one standard space (MNI152NLin2009cAsym) was performed through nonlinear registration with antsRegistration (ANTs 2.2.0), using brain-extracted versions of both the T1w reference and the T1w template. The following template was selected for spatial normalization: ICBM 152 Nonlinear Asymmetrical template version 2009c [32].

##### Functional Data Preprocessing

Results included in this manuscript come from preprocessing performed using fMRIPrep 1.4.0 ([33], RRID:SCR_016216), which is based on neuroimaging pipeline in python (Nipype 1.2.0) [34,35]. For each of the resting-state fMRI runs, the following preprocessing was performed. First, a reference volume and its skull-stripped version were generated using a custom methodology of fMRIPrep. The BOLD reference was then co-registered to the T1w reference using bbregister (FreeSurfer), which implements boundary-based registration [36]. Co-registration was configured with nine degrees of freedom to account for distortions remaining in the BOLD reference. Head-motion parameters with respect to the BOLD reference (transformation matrices, and six corresponding rotation and translation parameters) were estimated before any spatiotemporal filtering using mcflirt (FSL 5.0.9, [37]). BOLD runs were slice-time corrected using 3dTshift from AFNI 20160207 ([38]). The BOLD time-series (including slice-timing correction) were resampled onto their original, native space by applying a single, composite transform to correct for head motion and susceptibility distortions. The BOLD time-series were resampled into standard space, generating a preprocessed BOLD run in MNI152NLin2009cAsym space. Several confounding time-series were calculated based on the preprocessed BOLD/framewise displacement (FD), the spatial root mean square of the data after temporal differencing (DVARS), and three region-wise global signals. FD and DVARS were calculated for each functional run, both using their implementations in Nipype (following the definitions by [39]). The three global signals were extracted within the CSF, the WM, and the whole-brain masks. Additionally, a set of physiological regressors were extracted to allow for component-based noise correction (CompCor, [40]). Principal components were estimated after high-pass filtering of the preprocessed BOLD time-series (using a discrete cosine filter with 128s cut-off) for the two CompCor variants: temporal (tCompCor) and anatomical (aCompCor). tCompCor components were then calculated from the top 5% variable voxels within a mask covering the subcortical regions. This subcortical mask was obtained by heavily eroding the brain mask, which ensures it does not include cortical GM regions. For aCompCor, components were calculated within the intersection of the aforementioned mask and the union of the CSF and WM masks calculated in T1w space after their projection to the native space of each functional run (using the inverse BOLD-to-T1w transformation). Components were also calculated separately within the WM and CSF masks. For each CompCor decomposition, the k components with the largest singular values wereretained, such that the retained components’ time series were sufficient to explain 50% of variance across the nuisance mask (CSF, WM, combined, or temporal). As a final step, a high pass filter was applied to the fMRI time courses to linearly detrend the signal.

#### 2.1.6. Diffusion MRI

Diffusion weighted images were acquired using high resolution 3T MR scans at the St. Joseph’s Hospital, London, ON, Canada for controls and at the IRCCS SDN for patients. All processing steps for tractography reconstruction and generation of structural connectivity matrices were performed with MRTrix toolbox, version 3.0, considering anatomical information carried out from Desikan-Killiany atlas based Freesurfer parcellation for cortical regions, and estimates from FSLs FIRST tool for subcortical areas. To increase the biological accuracy of the reconstructions, processing steps taking advantage of the anatomically constrained tractography (ACT) framework [41] were performed. Two different streamlined reconstruction approaches, probabilistic and deterministic [42,43], were performed on a multi-shell, multi-tissue constrained spherical deconvolution fiber orientation distribution model [44,45]. Reconstruction constraints were maximum streamline length equal to 25 centimeters, streamline seeds belonging to segmented white matter, and one million reconstructed streamlines per subject. All streamlines were mapped to the parcellated image to produce a structural connectome, considering the number of streamlines as structural connectivity weight. In addition, FA derived from conventional diffusion tensor tractography at b-value 3000, were mapped across probabilistic and deterministic streamlines, providing FA matrices that can be used as a proxy for structural connections integrity.

### 2.2. Generalized Ising Model Simulations

The GIM was simulated using the normalized structural connectome obtained from controls as well as from patients. Simulations were performed starting with a random spin configuration of size 1xL (L = 84). The spin configuration was placed in contact with a thermal bath of temperature T. Each spin could be in only one of the two spin states (either up (+1) or down (−1)). Both the equilibrium spin configuration and the thermodynamic properties of the system change with respect to the temperature. Energy of this spin configuration can be calculated in the absence of an external magnetic field by Equation (1),

(1) $$E(x)=-\;\sum_{i,j}^{N}\;J_{ij}\;S_{i}S_{j}$$

where Jij is the coupling (introduced through the structural connectome) between the i^th^ and j^th^ regions, Si and Sj represent the spins of the i^th^ and j^th^ regions, respectively, and *n* = 84. The GIM simulations were performed for each subject/patient over a range of temperatures starting with a random spin configuration using the metropolis Monte Carlo algorithm [22]. This process was repeated ten times for each structural connectome to ensure that we captured different random spin configurations and the results were averaged over these ten simulations. Each simulation resulted in a time series for each of the spin sites, permitting the calculation of a simulated correlation matrix using Pearson’s correlation. Then, the simulated correlation matrices were compared with the empirical correlation matrices using the Mantel test within the group of controls as well as patients, and the results between the two groups were also compared. Dimensionality of the brain was calculated for all the data via the generalized Ising model simulations as explained in the Supplementary Materials: Dimensionality Calculations. MATLAB code developed in-house (in MATLAB 2018b) was used for all computer simulations.

### 2.3. Multimodal Connectograms

Structural and functional connectomes were synergistically inspected via a multimodal representation. A modified version of the visualization routine implemented in the open-source multimodal imaging brain connectivity analysis (MIBCA) toolbox ([46], e1078) was adopted to generate multimodal connectograms. In particular, the multimodal connectogram consisted of a circular depiction where the most external ring corresponded to the cortical and subcortical GM regions of the Desikan–Killiany atlas, the internal rings showed the t1, t2, and simulated functional data, from inside to outside, respectively, and lines internal to the circle reflected structural connectivity between each pair of GM regions. For functional data, the connectivity was represented as degree, i.e., the cumulative connections for each brain region. Average connectivity of each population (healthy participants, MCS, and UWS) was graphically depicted with multimodal connectograms.

## 3. Results

**Figure 1:**
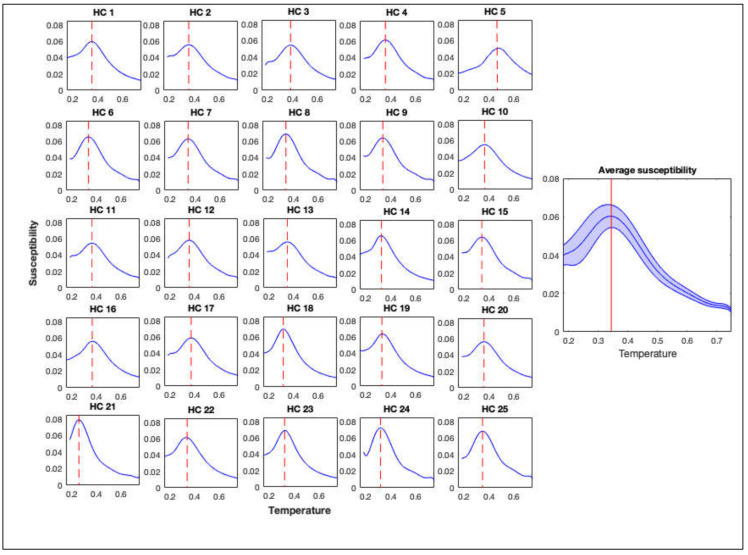
Magnetic susceptibility calculated from the generalized Ising model (GIM) for healthy controls (HC) (individual and average). Red dashed line and the solid line represent the critical temperature for the individuals and the average respectively.

**Figure 2:**
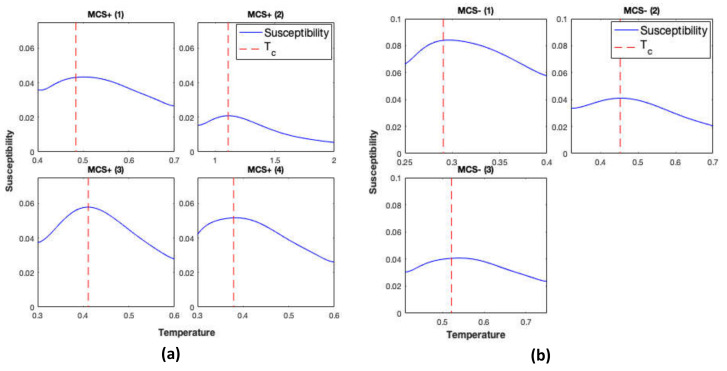

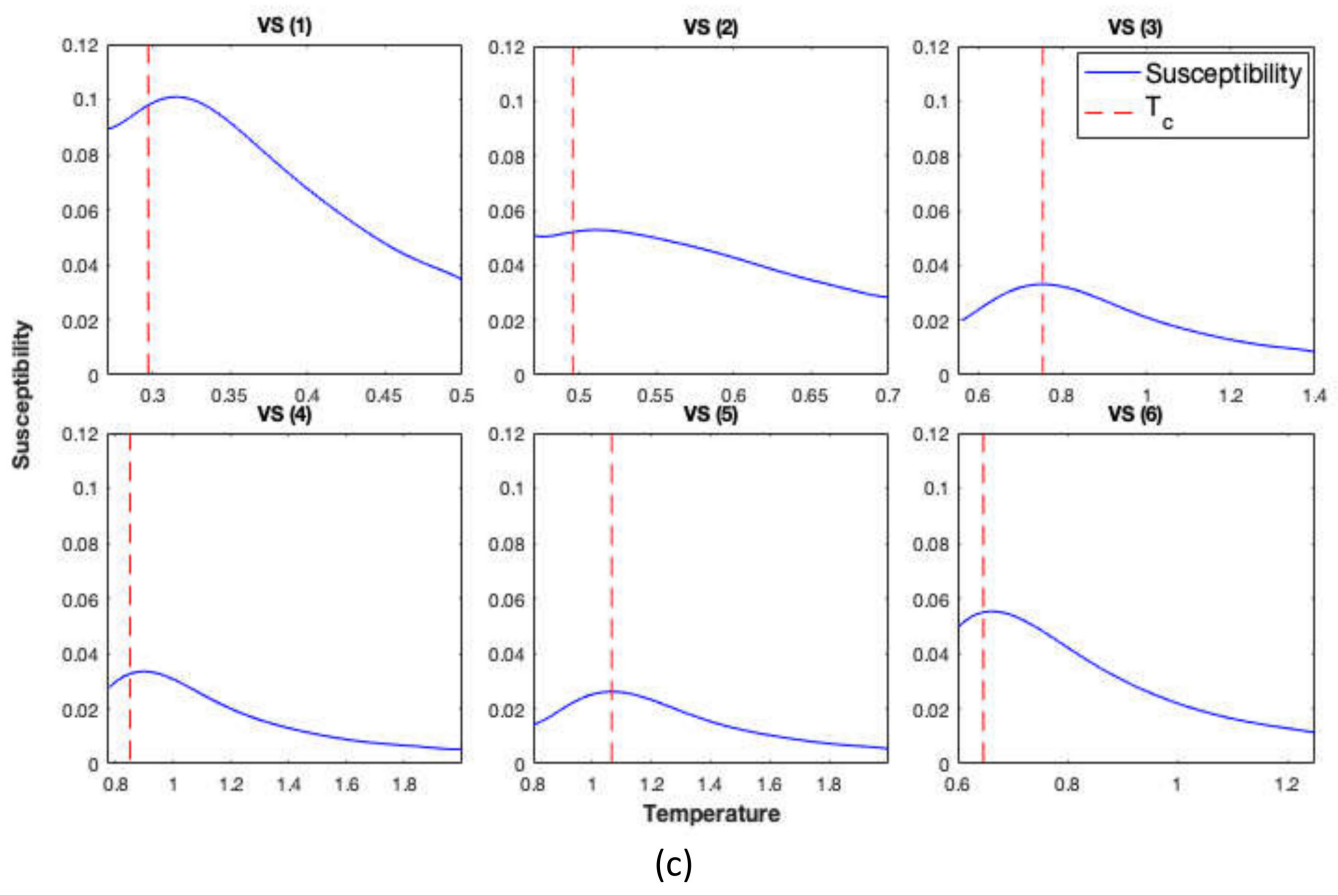
Magnetic susceptibility calculated from the GIM for disorder of consciousness (DoC) patients in minimally conscious state positive (MCS+) in panel (**A**), minimally conscious state negative (MCS−) in panel (**B**), and vegetative state (VS) states in panel (**C**). Red dashed line represents the critical temperature.

The GIM was simulated using structural connectomes of 25 controls and structural connectomes of 13 patients with DoC. Simulations were repeated using each structural connectome ten times. Averages (over the ten simulations) of the magnetic susceptibility of the GIM were calculated and are plotted as a function of the temperature in Figure 1 and Figure 2 for controls and DoC patients, respectively. Magnetic susceptibility was used to locate the critical temperature of the simulations.

**Figure 3:**
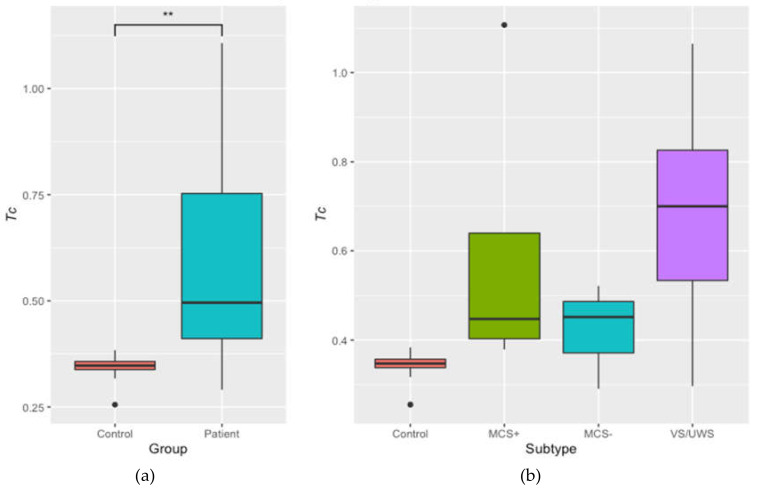
Illustration of the critical temperature (*Tc*) in controls and patients (**A**) for the average of controls and patients and (**B**) for the average of the controls and separate patient groups. ** *p* < 0.01.

Differences observed in the temperature ranges between controls and patients and even within the patients’ groups are observed as a result of the differences/alteration between the structural connectomes of patients in different stages compared to controls. We further examined the distribution of the critical temperature between these groups, and results are presented in Figure 3A. A Welch two sample *t*-test between the controls and patients showed that there is a significant difference of the critical temperature between the two groups *(t* (12.21) = −3.34, *p* = 0.006; Figure 3A). A one-way ANOVA indicated that there was a main effect of subtype (*F* (3,25) = 6.54, *p* = 0.002), with a difference between healthy controls and VS/UWS, however, this did not survive false discovery rate (FDR) correction (*t* (5.04) = −3.09, *p* = 0.162; Figure 3B).

**Figure 4:**
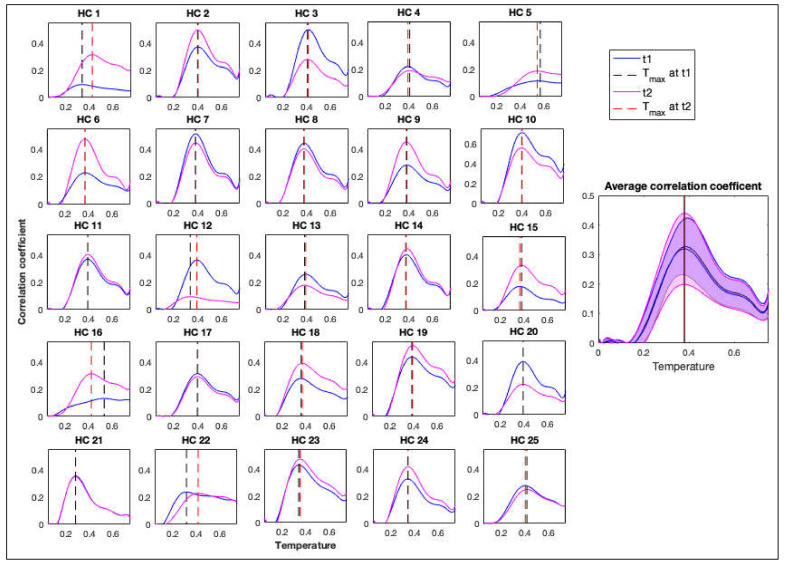
Correlation coefficients between the empirical and simulated functional connectivity matrices for healthy controls (HC) individually and as an average for scans t1 and t2, separated by 30 min. Black dashed line represents *Tc* for the first scan and the red dashed line represents *Tc* for the second scan.

**Figure 5:**
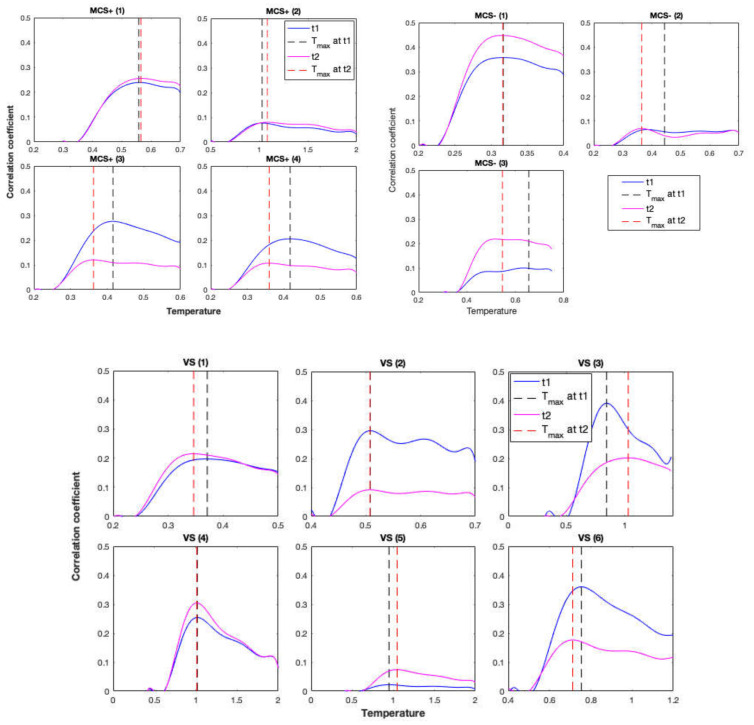
Correlation coefficients between the empirical and simulated functional connectivity matrices calculated by the Mantel test for DoC patients’ scans t1 and t2, separated by 30 min. Black dashed line represents *Tc* for the first scan and the red dashed line represents *Tc* for the second scan.

The correlation between the simulated and empirical functional connectivity was calculated using the Mantel test and is presented as a function of temperature in Figure 4 and Figure 5 for controls and patients, respectively. Correlations were calculated using empirical functional connectivity obtained from both t1 and t2 scans, which were acquired 30 min apart. These correlation measures were used to obtain T*, the temperature that maximizes the correlation between simulated and empirical functions.

**Figure 6:**
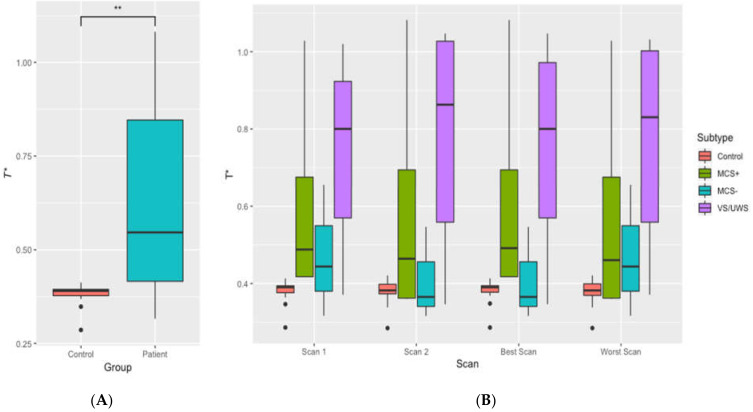
Illustration of T* in controls and patients. (**A**) for the average of controls and patients and (**B**) for the average of the controls and separate patient groups. Panel (**B**) also illustrates the T* values for two separate scans as well as the best and the worst scans. ** *p* < 0.01.

For controls, a pairwise *t*-test showed that there was no significant difference in T* between the two scans (*t * (24) = 0.58, *p* = 0.571). Patients’ T* values for the two scans also did not show a significant difference (*t* (12) = 0.09, *p* = 0.932) as shown in Figure 5. T* values are shown in Figure 6A,B for further comparisons. There was also a significant difference between T* values of the controls and patients (*t* (12.22) = −3.21, *p* = 0.007). Therefore, the representation of results hereafter includes the results for the average as well as the results for the best scan out of the two (with the highest correlation coefficient) for comparisons.

**Figure 7:**
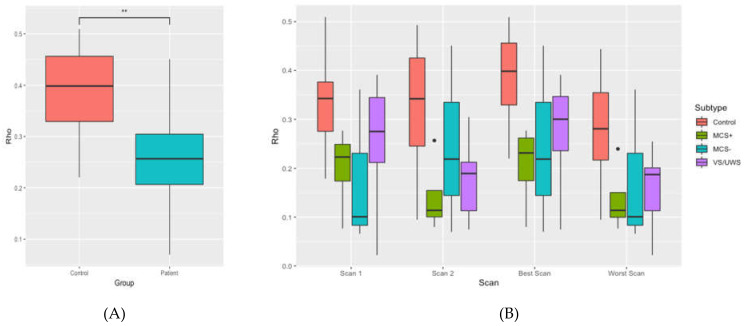
Correlation coefficients between the empirical functional connectivity and the simulated functional connectivity matrices for controls and patients (**A**) for the average of controls and patients and (**B**) for the average of the controls and separate patient groups. ** *p* < 0.01.

To further examine the correlations between empirical and simulated functional connectivity matrices, the correlation at *Tc* was plotted for controls and patients in Figure 7A,B, and a pairwise *t*-test showed that the correlation between empirical and simulated functional connectivity matrices were significantly different for controls and patients with (*t* (20) = 2.85, *p* = 0.009).

**Figure 8:**
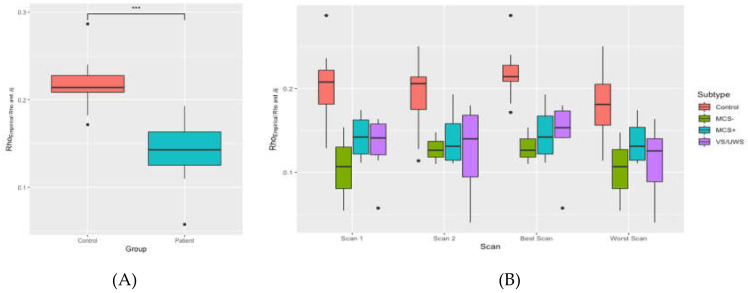
Correlation coefficients between empirical functional connectivity and empirical structural connectivity matrices for controls and patients (**A**) for the average of controls and patients and (**B**) for the average of the controls and separate patient groups. *** *p* < 0.001.

Additionally, to illustrate the improvements in the predictability as a result of introducing the generalized Ising model, Figure 8 shows the direct correlations between empirical functional and structural connectivity matrices. By comparing the (A) panes from Figure 7A,B, and Figure 8A,B, it can be observed that the introduction of the GIM to find a relationship between the structure and the function of the brain improves the correlations from approximately 0.2 to 0.4 for controls and from 0.15 to 0.25 for the patients’ population.

**Figure 9:**
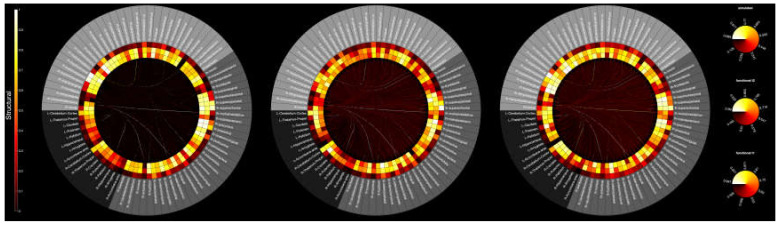
Multimodal representation of structural and functional connectivity. The outermost circle corresponds to subcortical (black) and cortical (dark grey for left, light grey for right) brain structures (Desikan–Kiliany atlas) used to derive structural and functional connectomes. The edges inside each circular graph depict structural connectivity between each pair of gray matter (GM) regions, whereas the rings refer to t1, t2, and simulated functional data, from inside to outside respectively. From left to right: average of normalized connectomes for healthy participants, MCS. and UWS.

Next, to compare the empirical functional and structural connectivity and simulated functional connectivity across brain regions, multimodal connectograms were plotted in Figure 9. The multimodal connectogram representation showed the correlations of both empirical and simulated functional data. In particular, the average connectograms reflected a similar trend of functional connectivity between empirical and simulated data.

**Figure 10:**
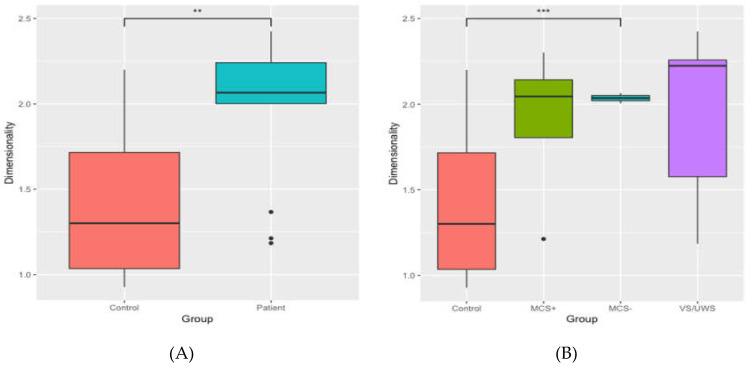
Dimensionality of the controls and the patients (**A**) for the average of controls and patients and (**B**) for the average of the controls and separate patient groups. *** *p* < 0.001, ** *p* < 0.01.

Finally, dimensionality was calculated for controls and patients and plotted in Figure 10. A Welch two sample *t*-test indicated that the dimensionality of controls and patients significantly differed (*t* (26.47) = −3.26, *p* = 0.003). A one-way ANOVA showed a significant effect of subtype (*F* (3,25) = 3.30, *p* = 0.037), and post-hoc *t*-tests indicated that MCS− patients had a significantly higher dimensionality than controls (*t* (15.68) = −5.34, *p* < 0.001).

## 4. Discussion

The present study examined whether the GIM can be extended to predict functional connectivity of the brain in patients with DoC, who often have widespread structural brain damage. We measured the empirical correlation between regions of the brain in 25 healthy controls and 13 patients with DoC and also created simulated correlations between regions using the structural connectivity of each participant using the GIM. These simulations were performed at various temperatures (constant *T*), in order to identify the point at which the system went from ordered to disordered (*Tc*). We then used this parameter to find the temperature at which the correlation between empirical and simulated data was highest (T *). From this, we calculated the dimensionality of the system for both patients and controls.

Dimensionality was found to be higher in patients than in controls, indicating that information decays faster in the brains of patients with DoC than in healthy brains. That is, with increasing distance between two regions, the correlation between two regions decreases faster in patients than in controls. Because we saw a faster rate of decay in patients across the same distance in each group, it suggests that this difference arises from pathology in something other than the brain’s structural connections. This highlights the importance of taking both structure and function into account within the same model; here, we found that the reduction of functional connectivity goes beyond what can be explained by the reduction of structural connectivity.

*Tc* is the point at which the system flipped from ordered to disordered. *Tc* is directly controlled by the structural connectivity of the participant; the significantly lower *Tc* in controls compared to patients implies a difference at the structural level. It has been previously shown that *Tc* increases with increasing sparsity in the data [22]. The group differences in *Tc* thus may reflect greater sparsity in the structural connections of the brain of patients with DoC. When we examined differences by patient sub-group, however, we found no statistically significant differences. The lack of differences could be due to the small sample size for each patient group, leading to large amounts of variance, which can be seen in Figure 3. The fact that patients have different aetiologies likely contributed to the variance as well; although the patients in each group have the same diagnosis, the specific damage is unique to each patient. Numerically, however, each patient group showed higher *Tc* than controls, and as a group this difference reached significance, suggesting that healthy brains are more connected than those of patients with DoC.

An important implication of these results is whether the GIM is able to successfully model the brain of individuals with DoC. The effectiveness of the GIM can be improved by using the structural connectivity of the brain to weight the model’s connections. If the pathological activity observed through fMRI in patients with DoC was purely the result of damaged connections, then there would be little concern that the model may not generalize to patient groups. However, we know that there are a number of issues that can cause pathological brain function that do not rely on the connections in the brain being damaged, such as metabolic processes through astrocytes or gliosis, neuronal damage, and thalamic damage [47,48]. While we observed differences in the model parameters between healthy controls and patients, the model was successfully able to improve prediction of brain function beyond what structure can offer alone. That we saw improvements in correlation levels between the empirical and simulated data not only in controls but also in patients indicates that the GIM is generalizable to patient populations, including those with highly damaged brain structure.

Another practical implication of the present study relates to multiple resting-state fMRI acquisitions. In efforts to detect consciousness in patients, it is especially important to ensure that the patients, if they are conscious, are awake and aware at the time of testing. Despite there being protocols for ensuring that the patient is maximally conscious (as much as their disorder permits), it is often impossible to confirm that they are indeed awake and aware at the time of the scan. Additionally, patients often move during a scan, lowering the quality of the data. Here, we were able to improve the overall correlation between our empirical and simulated data by acquiring two scans per patient and using that which gave the highest correlation on a subject-wise basis. As can be seen in Figure 7, by using the best by-subject scan, correlation measures improved in all four groups (i.e., VS/UWS, MCS−, MCS+, healthy controls). These results highlight the importance of having good quality data, especially in patient groups, which might require multiple runs. This would be especially important in task-based data, as patients in a minimally conscious state will have fluctuating levels of wakefulness and awareness. These results underscore the importance of not making assumptions or drawing conclusions about a patient’s level of consciousness based on a single scan session. From this, we recommend that DoC patients are scanned at least twice [49], however, more research is needed to determine the gold standard.

We were also able to replicate the results of a previous study [22], confirming that the GIM can be used to simulate data in healthy populations. Although this was not the focus of the present study, these results are important as they show that the model is generalizable and replicable within healthy individuals. Future research will need to replicate the present DoC results in an independent sample of patients to confirm that they are reproducible in such populations. This is likely to be a more difficult task, as each patient has suffered a unique pattern of damage, which leads to a highly variable sample.

Several questions remain. First, could dimensionality, *Tc*, or T* be used in the future to determine a patient’s level of consciousness? This seems likely; there were several reliable differences in the GIM parameters between healthy controls and patients with DoC. Although DoC subtypes did not show differences, the numerical trend suggests that future research with larger samples may find these parameters to be predictive of the patient’s state of consciousness. While this method shows promise, at the moment, the interpretation of the GIM parameters should be combined with other measures to assess the level of consciousness, such as command-following fMRI tasks [46] and behavioural measures like the CRS and Glasgow coma scale (GCS). Second, can these measures be used to identify patients whose condition is likely to improve? While the present study did not examine this question, future research should apply this methodology to longitudinal data. It is possible that the GIM has predictive power, however, this is an empirical question that must be tested.

## 5. Conclusions

For the first time, we demonstrate that the GIM is applicable to modelling brain function in patients with DoC. The present study also applied a novel approach to calculate the brains’ dimensionality in DoC patients via the GIM. Indeed, the cross-sectional investigation on 13 patients with DoC demonstrated that dimensionality differed between diagnostic groups (i.e., VS/UWS, MCS−, and MCS+) and could distinguish them from healthy controls. This approach could be useful for determining the level of consciousness We acknowledge that the low number of patients and the heterogeneity in clinical diagnosis and etiology is the principal limitation of this study. However, it is important to underline that this sample size is comparable or higher to those reported in available published neuroimaging studies on DoC patients because of issues in acquiring functional neuroimaging in such non-collaborative and complex patients. Although the sample size of the present study did not allow for any generalization, we hope that our preliminary results can serve as a starting point for devising further multicenter research on larger homogeneous samples in order to standardize this innovative method and to investigate its possible contribution to current procedures and guidelines for diagnosis and prognosis [12,50].

## Figures and Tables

**Table 1:** Demographic, anamnestic, and clinical characteristics of patients at PET/MRI scan.

Pt	Age at Onset	Sex	Aetiology	Time Post-Onset (Months)	Clinical Diagnosis	CRS-R Total Score (Subscales)	Conscious Behaviour
1	34	F	Anoxic	8	VS/UWS	7 (2 + 1 + 0 + 2 + 0 + 2)	NA
2	58	F	Vascular	10	MCS+	11 (3 + 1 + 4 + 1 + 0 + 2)	Reproducible command following; object manipulation
3	57	M	Anoxic	6	MCS+	12 (3 + 1 + 5 + 1 + 0 + 2)	Reproducible command following; automatic motor response
4	18	M	Traumatic	3	MCS+	11 (3 + 3 + 2 + 1 + 0 + 2)	Reproducible command following; visual pursuit
5	53	M	Anoxic	7	VS/UWS	6 (1 + 0 + 2 + 1 + 0 + 2)	NA
6	70	M	Vascular	3	VS/UWS	7 (1 + 1 + 2 + 1 + 0 + 2)	NA
7	44	F	Vascular	10	MCS−	7 (1 + 3 + 0 + 1 + 0 + 2)	Visual pursuit
8	38	M	Anoxic	6	VS/UWS	4 (2 + 0 + 1 + 0 + 0 + 1)	NA
9	73	F	Vascular	5	VS/UWS	6 (1 + 1 + 1 + 1 + 0 + 2)	NA
10	48	M	Traumatic	3	MCS−	10 (2 + 3 + 2 + 1 + 0 + 2)	Visual pursuit
11	37	M	Vascular	3	MCS+	21 (4 + 5 + 5 + 3 + 1 + 3)	Consistent command following; object recognition; automatic motor response; intelligible vocalization
12	24	M	Anoxic	2	MCS−	8 (1 + 2 + 2 + 1 + 0 + 2)	Visual fixation
13	35	M	Traumatic	4	VS/UWS	7 (1 + 1 + 2 + 1 + 0 + 2)	NA

Note: F = female; M = male; CRS-R = coma recovery scale-revised; VS/UWS = vegetative state/unresponsive wakefulness syndrome; MCS− = minimally conscious state minus; MCS+ = minimally conscious state plus. NA = not applicable. The conscious behaviours assessed by the coma recovery scale subscales are marked in bold and described in the additional column.

## Supplementary Materials

The following are available online at https://www.mdpi.com/2077-0383/9/5/1342/s1, Figure S1: Flow chart of patient selection in each step of the study, Document: Dimensionality Calculation.
