# Supplementary material for: Consciousness and the Dimensionality of DOC Patients via the Generalized Ising Model
Source: J Clin Med. 2020 May 4;9(5):1342. doi: 10.3390/jcm9051342 (PMC7290966; doi:10.3390/jcm9051342)
Supplement: Supplementary file 1 [file jcm-09-01342-s001.pdf]

Supplementary material:

Figure S1: Flow chart of patient selection in each step of the study

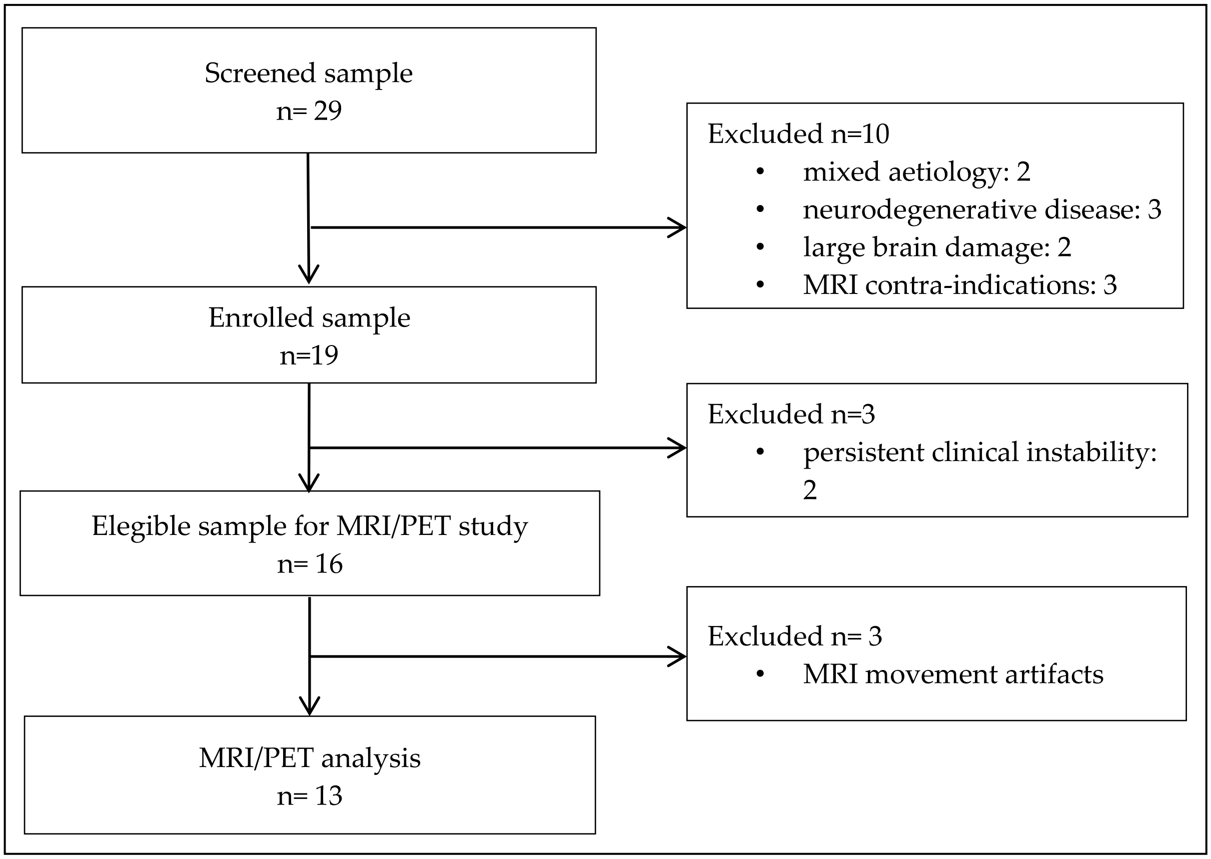

Dimensionality calculation

Calculating the Dimensionality of a System Using the Results of Generalized Ising Model Simulations

Normalized structural connectivity (J_{ij}) of the system/network is used in the generalized Ising model simulations which results in the time series for each node of the network for a given number of time points. Pearson's' correlation between these time series was calculated which results in a N x N symmetric correlation matrix (where N is the number of nodes in the network/ number of sites). This process was carried out for a range of temperatures.

Calculating the correlation function, [G(r)]

Correlation function per temperature illustrates how does the correlation changes with respect to the distance between the nodes. To calculate the correlation function, first the distance between the nodes were defined using the structural connectivity matrix as $^1/_{J_{ij}}$. Then the correlation was grouped such that the correlation at a particular distance is the average of all the correlations at that distance. This results in a correlation versus distance plot (Fig. 1) for each temperature.

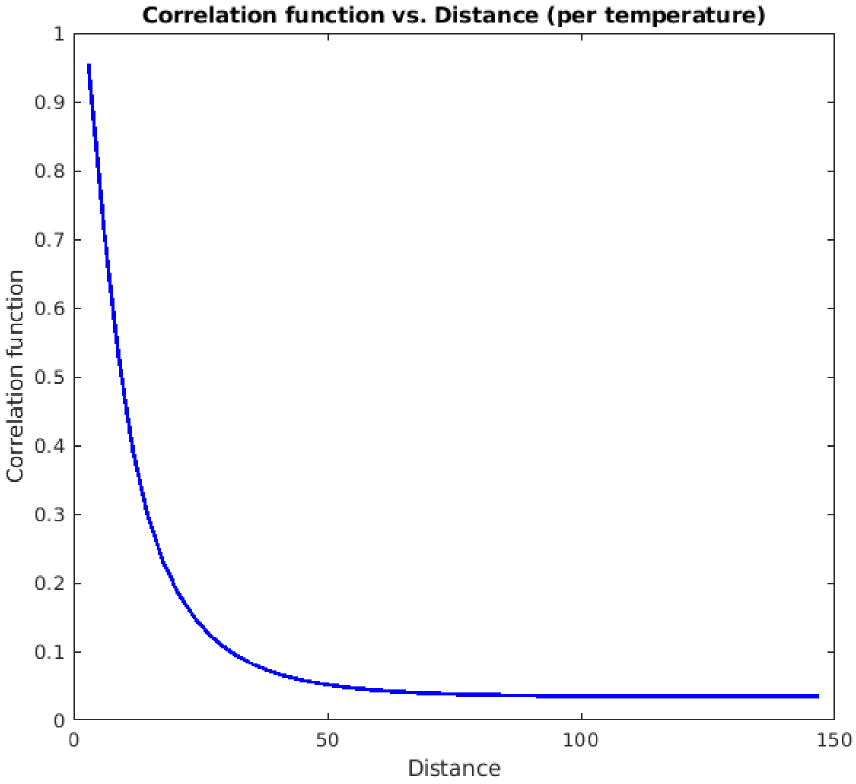

Figure 1: Correlation functions as a function of distance at one temperature for the generalized Ising model simulated using the structural connectome (average over 69 subjects) of human brain (84 ROIs)
Correlation function can be explained using Equation 1 for any temperature (1, 2) except the critical temperature. This equation simplifies to Equation 2 at the critical temperature since the correlation length (ξ) becomes infinite.

$$G(r) \approx \frac{\exp\left(\frac{-r}{\xi}\right)}{r^{d-2+\eta}} = \frac{\exp(\frac{-r}{\xi})}{r^{P_0}} \quad (1)$$

$$G(r) \approx \frac{1}{r^{P_0}} \quad (2)$$

Calculating the dimensionality

From Ornstein-Zernike theory, another equation was developed to explain the correlation function at temperatures greater than the critical temperature. It is presented in Equation 3 (2, 1).

$$G(r) \approx \frac{\exp\left(\frac{-r}{\xi}\right)}{r^{\frac{d-1}{2}}} \quad (3)$$

By equating the power of r in the denominator of Equation 1 and 3,

$$P_0(T > T_c) = \frac{d-1}{2} \quad (4)$$

$$\therefore\ d = 2P_0 + 1 \qquad (5)$$

Procedure: After calculating G(r) for all the temperatures, Equation 1 was fitted and P_0 and ξ per temperature were obtained from the fitting. Then P_0 values for $T > T_c$ were separated and averaged to obtain one P_0 value. Finally using the calculated P_0 values and Equation 5, dimensionality of the system was calculated.

References

1. Stanley, H Eugene. Phase transitions and critical phenomena. ,Clarendon Press, Oxford, 1971.

2. Landau, David P and Binder, Kurt. A guide to Monte Carlo simulations in statistical physics. ,Cambridge university press, 2014.
